# Multi-level characteristics recognition of cancer core therapeutic targets and drug screening for a broader patient population

**DOI:** 10.3389/fphar.2023.1280099

**Published:** 2023-11-23

**Authors:** Yangguang Su, Ying Wang, Zhuo Qu, Jiaxin Liu, Xuekun Ren, Denan Zhang, Xiujie Chen

**Affiliations:** ^1^ Department of Pharmacogenomics, College of Bioinformatics and Science Technology, Harbin Medical University, Harbin, China; ^2^ School of Mathematics, Harbin Institute of Technology, Harbin, China

**Keywords:** synthetic lethality, targeted therapy, multi-omics data, core therapeutic targets, targeted drug screening

## Abstract

**Introduction:** Target therapy for cancer cell mutation has brought attention to several challenges in clinical applications, including limited therapeutic targets, less patient benefits, and susceptibility to acquired due to their clear biological mechanisms and high specificity in targeting cancers with specific mutations. However, the identification of truly lethal synthetic lethal therapeutic targets for cancer cells remains uncommon, primarily due to compensatory mechanisms.

**Methods:** In our pursuit of core therapeutic targets (CTTs) that exhibit extensive synthetic lethality in cancer and the corresponding potential drugs, we have developed a machine-learning model that utilizes multiple levels and dimensions of cancer characterization. This is achieved through the consideration of the transcriptional and post-transcriptional regulation of cancer-specific genes and the construction of a model that integrates statistics and machine learning. The model incorporates statistics such as Wilcoxon and Pearson, as well as random forest. Through WGCNA and network analysis, we identify hub genes in the SL network that serve as CTTs. Additionally, we establish regulatory networks for non-coding RNA (ncRNA) and drug-target interactions.

**Results:** Our model has uncovered 7277 potential SL interactions, while WGCNA has identified 13 gene modules. Through network analysis, we have identified 30 CTTs with the highest degree in these modules. Based on these CTTs, we have constructed networks for ncRNA regulation and drug targets. Furthermore, by applying the same process to lung cancer and renal cell carcinoma, we have identified corresponding CTTs and potential therapeutic drugs. We have also analyzed common therapeutic targets among all three cancers.

**Discussion:** The results of our study have broad applicability across various dimensions and histological data, as our model identifies potential therapeutic targets by learning multidimensional complex features from known synthetic lethal gene pairs. The incorporation of statistical screening and network analysis further enhances the confidence in these potential targets. Our approach provides novel theoretical insights and methodological support for the identification of CTTs and drugs in diverse types of cancer.

## Highlights

• A strategy for the identification and drug screening of core therapeutic targets (CTTs) is proposed to address the narrow population of beneficiaries of targeted cancer therapies and the vulnerability of patients to drug resistance.
• A comprehensive character extraction and representation approach based on big data from multi-omics biology was established at different levels such as gene expression, epigenetic and genomic variation and different perspectives such as expression regulation and post-transcriptional regulation. The fundamental characteristics of the synthetic lethal mechanism of cancer are further revealed.
• The predictive model based on synthetic lethal mechanisms proposed in this study can identify novel targets that play a central role in multiple cancer-related disease pathways at the level of gene expression, genetic and genomic variation, and screen for potential therapeutic agents in different dimensions of gene transcriptional regulation and post-transcriptional regulation, greatly expanding the existing theoretical and technical approaches to target identification and drug screening in targeted cancer therapy.
• Case studies of the identification of CTTs and the discovery of corresponding targeted therapeutics in colorectal, lung and kidney cancers, as well as results from other research literature, databases and wet experiments, suggest that our target and drug identification models are quite generalizable. The nature and biology of the cancers identified by the CTTs model are widely present in different cancer types and have great potential for application and reference value for therapeutic target discovery across cancer types.

## 1 Introduction

Molecular targeted therapy, which specifically destroys cancer cells through biological mechanisms such as inhibition of tumor growth, metastasis, angiogenesis, and promotion of apoptosis, has rapidly become the first-line clinical treatment for cancer due to its high efficacy and low toxicity, significantly improving the survival time of cancer patients (Bennouna et al., 2019; Sveen et al., 2020; Chan et al., 2022; Hussain et al., 2022; Jaaks et al., 2022; Tan and Tan, 2022). Unfortunately, targeted therapeutic approaches target certain specific genetic mutations in cancer patients, which has resulted in a scarcity of clinical candidate therapeutic targets and targeted therapeutic drugs. The small range of populations benefiting from targeted therapies (Saito et al., 2018) and the acquired resistance of patients after long-term application are also major challenges in current clinical practice (Saito et al., 2018; Cabanos and Hata, 2021; Zugazagoitia and Paz-Ares, 2022).

Synthetic lethality (SL) is a phenomenon in which simultaneous repression of two non-lethal genes results in cell death while only one of the genes is repressed and the cell still survives. Synthetic lethality offers the potential for precision targeting of incompetent gene mutations in cancer cells (Bortlikova et al., 2019). Targeted therapies based on SL mechanism increase the number of candidate targets on the one hand and solve the challenge of acquiring drug resistance on the other hand. When genomic defects or compensatory pathways are combined with targeted therapy, it has significant antitumor activity (Lopez and Banerji, 2017). Precision therapies based on the SL mechanism have been gradually applied to cancer, such as PARP inhibitors (Lord and Ashworth, 2017), Farmer and Bryant et al. proposed a new strategy of PARP inhibitors of BRCA1 or BRCA2 for the treatment of breast cancer patients with BRCA mutations (Bryant et al., 2005; Farmer et al., 2005) and Taylor et al. proposed a treatment strategy for patients with locally advanced and metastatic breast cancer with HER2 mutations (Taylor et al., 2021). At present, the computational method of SL pairs recognition is mainly based on transcriptional spectrum data (Apaolaza et al., 2017) and machine learning (Sinha et al., 2017; Liu et al., 2018), but the effectiveness of prediction results needs to be improved. In addition, it is usually possible to identify SL pairs through large-scale gene knockout experiments (Dhanjal et al., 2017), but it is time-consuming and expensive. These factors limit the discovery of SL pairs, which leads to the scarcity of SL target candidates and targeted therapeutic drugs.

Therefore, we propose to overcome this problem by identifying “core therapeutic targets" (CTTs) that is the hub genes in the network. A core therapeutic target is a potential therapeutic target that has a central position in the process of performing therapeutic effects through multiple related biological processes. Due to the large number of mutations in genes that are synthetic lethal partners of CTTs, identifying and targeting CTTs can maximize the therapeutic effect, reduce drug resistance and expand the potential beneficiary population. In addition, cancer-related specific regulatory signals are often important contributors to its development and progression. Potential targets are regulated at multiple levels, which not only reflects the complexity of the biological processes in which they are involved, but also provides a wider range of possibilities for new drug development. We therefore analyzed the regulatory factors of CTTs such as miRNAs, lncRNAs and super-enhancers, and finally identified CTTs in CRC as new potential drug targets. These regulatory characteristics provide the possibility for new potential therapeutic drug targets in the synthetic lethal relationship. In this study, we propose a new statistical machine learning and network analysis screening framework for cancer to determine new potential therapeutic targets and further screen relevant targeted therapeutic agents or compounds according to the SL mechanism of multidimensional biological data of cancer. The results for lung cancer and renal cell carcinoma confirm that the strategy proposed in this study can also be applied to targeted therapeutic target identification and drug screening for other cancers. Our approach provides new theoretical and methodological insights into the identification of CTTs and therapeutic drugs with broad effects in different cancer types.

## 2 Methods and materials

**FIGURE 1:**
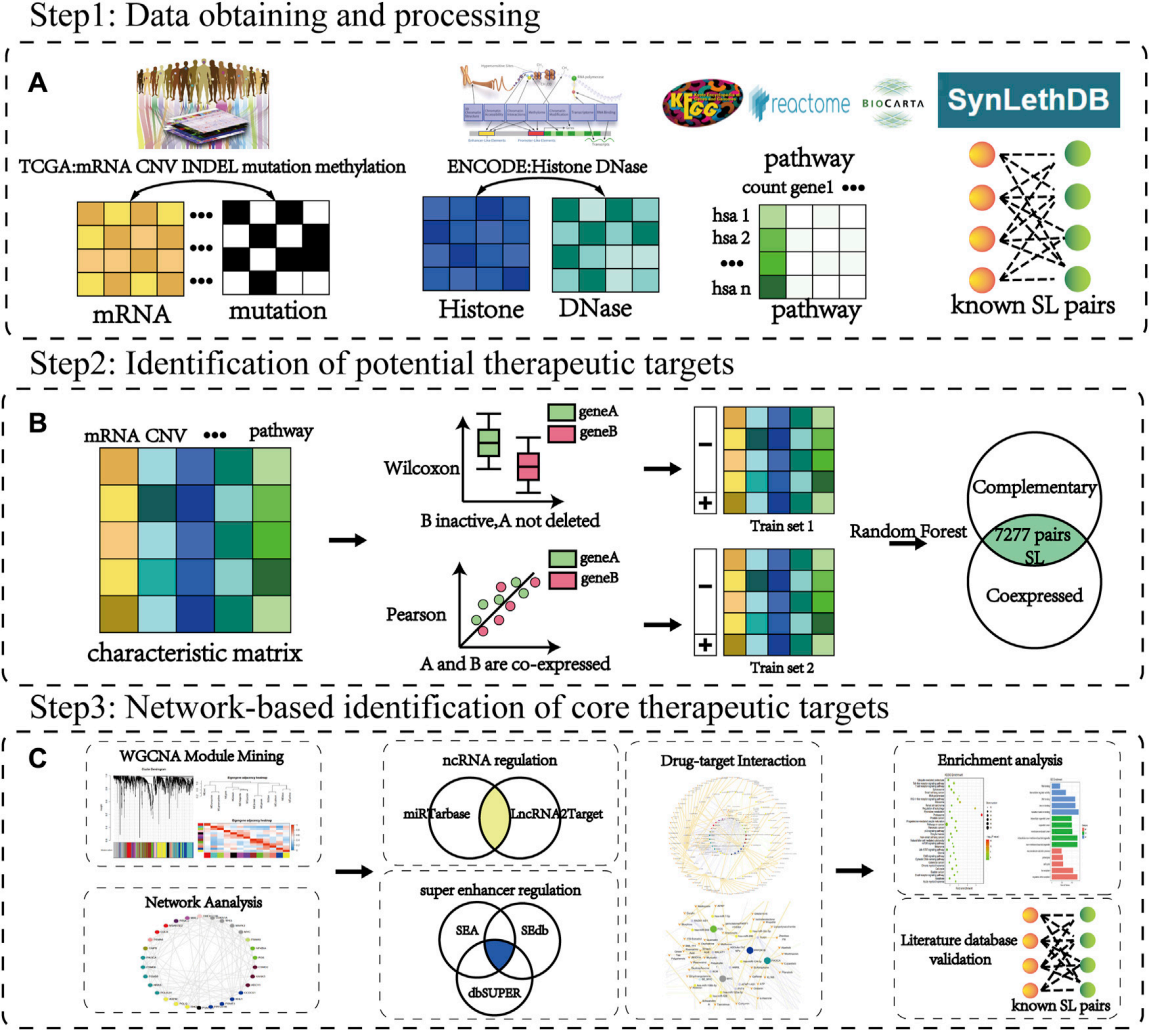
The workflow of CTTs identification and corresponding therapeutic drug screening. **(A)** Step 1: Data source. Cancer data is mainly from TCGA, ENCODE, KEGG, Reactome, Biocarta database, and the SL pairs from the SynLethDB database. **(B)** Step 2: Identification of potential therapeutic targets. The characteristic matrix is the data of each omic containing SL pairs. The random forest classifier model is constructed based on the characteristic samples of the SL compensation mechanism and co-expression mechanism obtained from the rank sum test (Wilcoxon) and correlation test (Pearson), respectively. **(C)** Step 3: Network-based identification of core therapeutic targets (CTTs). The WGCNA and network analysis are used to filter hub modules and hub nodes. Through further recognition of super-enhancer and ncRNA regulation, we can verify the target and screen its targeted therapeutic drugs.

In this study, we established a workflow to identify new therapeutic targets (CTTs) based on multi-omics data to extract mechanism-defined interaction and regulatory features between SL genes in response to the challenges of the small number of beneficiary populations and susceptibility to drug resistance currently faced in targeted cancer therapy. Following the biological characteristics, such as SL genes with functional compensatory features and co-expression among them, we constructed machine learning models for predicting corresponding mechanisms from the multi-omics features of known SL pairs, respectively. To improve the accuracy of identified targets and reduce false positives, we combined the results of both types of predictions as identified SL genes. Finally, among these identified SL genes, we identified those genes with more potential SL partners as potential therapeutic targets for CTTs by network analysis and further screened the corresponding potential therapeutic drugs. The specific workflow of the article is shown in Figure 1.

### 2.1 Multi-omics data and characterization of SL

Multi-omics characteristics including mRNA, CNV, mutation, indel, methylation, histone, DNase and pathway were used to characterize SL genes in cancer. Among them, gene expression and mutations, as visual representations affecting gene function, are the main characteristics currently used in the computational prediction of SL interactions (Jerby-Arnon et al., 2014; Ryan et al., 2014; Dhanjal et al., 2017; Horlbeck et al., 2018). Epigenetic factors and proteins also have important regulatory roles in gene expression and its function, where DNA methylation decreases gene activity (Holliday and Pugh, 1975; Moore et al., 2013) and DNase regulates gene expression by affecting chromatin accessibility (Bernardi and Boyer, 1971; Zhang et al., 2014). Therefore, we obtained the expression profile, mutation, copy number variation and DNA methylation data of 182 colorectal cancer (CRC) samples from TCGA (Tomczak et al., 2015) database. Human hg19 reference genome 19444 genes were from UCSC (Casper et al., 2018) database. Histone data and DNase data of CRC samples were extracted from ENCODE (Davis et al., 2018) database. Paired SL genes are often involved in biological processes with high relevance (Ikui et al., 2012; Costa-Cabral et al., 2016; Wong et al., 2016), therefore the functional pathways they are involved in were used as one of the descriptive features of SL in this study. Pathway data were obtained from KEGG (Kanehisa et al., 2017), Reactome (Fabregat et al., 2018), and BIOCARTA (Nishimura, 2001) databases. Known 16916 SL interactions were downloaded from SynLethDB (Guo et al., 2016) database. For the multi-omics data of CRC, we matched each sample of CRC patients according to the gene of the human reference genome hg19. Then for the missing value, we supplemented it according to the mean value of the gene in other CRC samples, and for the mutation data, we supplemented the missing value with 0. The classifier model characteristic is shown in Supplementary Table S1.

### 2.2 Identification workflow of core therapeutic targets (CTTs) in cancer

#### 2.2.1 A machine learning-based framework for identifying potential SL pairs

This study identifies CTTs based on SL mechanisms in cancer cells. To identify CTTs that may play a therapeutic role across multiple biological functions and different mechanisms, we developed a two-step workflow. First, we screened currently known SL interactions for their biological properties at multiple omics levels to construct a model for identifying potential SL genes in cancer. This is followed by further screening of potential SL genes to identify CTTs.

A common feature is the compensatory relationship between SL gene pairs. The simultaneous functional inactivation of SL paired genes leads to cancer cell death, while the inactivation of one gene allows the other gene to become essential for cell survival and thus produce overexpression to compensate for the loss of function of the inactivated gene product (Beroukhim et al., 2010; Barretina et al., 2012). The Wilcoxon rank sum test was used to retain the significantly compensated pairs (*p*-value<0.01) according to DAISY in our study (Ryan et al., 2014).

Another common feature is the synergistic relationship between SL gene pairs. Many studies providing experimental confirmation of SL pairs suggest that SL pairs in cancer cells are more likely to be involved in biological processes that are closely related to each other and therefore both are usually co-expressed (Kelley and Ideker, 2005; Costanzo et al., 2010; Ryan et al., 2014). Therefore, we calculated the Pearson correlation coefficient to retain the significantly co-expressed pairs (*p*-value<0.05 and R > 0.5) according to DAISY (Ryan et al., 2014). We then used the known SL pairs screened by Wilcoxon and Pearson statistics as positive samples, the randomly selected gene pairs from the hg19-encoding gene pairs and removing known positive sample pairs were used as the negative sample sets to construct the identification models separately (positive:negative = 1:20).

Based on the experimentally confirmed SL pairs screened by the above mechanism that exhibit classification features on multiple omics, we constructed a recognition model using the randomForest machine learning method (R package randomForest with default parameters) and used the intersection of the recognition results obtained from different positive sample sets as the potential SL pairs identified in CRC (Supplementary Table S6).

#### 2.2.2 Network-based identification of core therapeutic targets (CTTs)

By integrating the consensus part of the predictions of the above models with different SL mechanisms, the potential SL pairs were obtained. A weighted gene co-expression correlation network is constructed using normalized gene expression data and known functional phenotypes. Genes that are intrinsically related are grouped into different modules according to the topology of this network, and the gene expression data in each module are clustered and analyzed to investigate the relevance of each internal gene and the different modules to processes in cell biology.

Consensus SL pairs from the results of potential target prediction models for CRC were used to construct a network describing the relationships between target genes using Cytoscape software. By analyzing the relationship between these potential therapeutic targets in the network, the top 1% of hub nodes in each module were identified as functionally relevant candidate “core therapeutic targets” (CTTs). By using these candidate CTTs, which are involved in multiple SL processes, as therapeutic targets, the corresponding inhibitor drugs can act on multiple disease-related pathways, thus overcoming the low applicability of the existing targeted drug population and the tendency to develop drug resistance.

### 2.3 Screening of potential targeted therapeutic drugs

At present, there are only a few gene targets and targeted therapeutic drugs for CRC in clinical practice, such as KRAS, NRAS, dMMR, MSI-H, BRAF, HER2, NTRK, and Cetuximab, Bevacizumab, Trastuzumab. The small number of these poses a significant challenge to CRC treatment, both in terms of low patient coverage and susceptibility to drug resistance. Computational research in the identification of new therapeutic targets for CRC based on SL mechanism and the subsequent discovery of new potential therapeutic drugs is one of the most promising drug discovery tools available, although it is often plagued by high false positive rates and inadequate therapeutic efficacy of the identified targets or drugs. To address these issues, in this study we not only propose an innovative approach to identifying CTTs with clear biological mechanisms to identify drug targets that act in multiple biological pathways. We also aim to broaden the drug population and reduce drug resistance by screening compounds that inhibit targets at multiple levels, including target binding, transcriptional regulation and post-transcriptional levels.

#### 2.3.1 Target analyzing and screening at the regulatory level

The complexity of the biological processes in which potential targets are regulated at multiple levels not only reflects the complexity of the biological processes in which they are involved but also opens up a wider range of possibilities for the development of new drugs. This study therefore furthers analyses and screens the CTTs candidate nodes identified in the network analysis at the level of transcriptional regulation and the level of post-transcriptional regulation. This was done based on the assumption that a more valuable target for cancer therapy should play a biological role at multiple levels of consideration. To this end, in addition to considering the existence of targeting relationships between targets and potential inhibitory molecules, we also mapped super-enhancers from the dbSUPER (Khan and Zhang, 2016), SEA (Chen et al., 2020), and SEdb (Jiang et al., 2019) databases and non-coding RNA regulatory data from the miRTarbase (Huang et al., 2020), LncRNA2Target (Cheng et al., 2019) to the CTTs candidate node correlation network constructed in this study, and ultimately identified CTTs in CRC as new potential drug targets by analyzing the regulatory impact of super-enhancers and non-coding RNA levels on CTTs candidate nodes.

#### 2.3.2 Durg-target interaction

Based on currently known target genes that are regulated at the super-enhancer and non-coding RNA levels, literature, and database searches were used to identify drugs that target the genes themselves or target gene regulatory elements, respectively, to establish a drug-target interaction network for CTTs, thus providing a viable reference for drug design or achieving drug repositioning in CRC.

### 2.4 Validation of results

In this study, the accuracy of the classifier model was evaluated by calculating the AUC values of the prediction model based on a tenfold cross-validation approach, and further, GO ontology analysis and KEGG pathway enrichment analysis of the predicted target genes using DAVID (Dennis et al., 2003) to validate the function of the prediction results. Finally, we also validated the prediction results by the available literature and database content. The case studies of potential CRC therapeutics obtained based on the predictions of this study also provided an additional level of support for the accuracy of our prediction results.

## 3 Results

### 3.1 Identification of synthetic lethal targets in CRC

To identify clinical candidate CTTs for targeted therapy, we first screened compensatory expression genes and co-expression propensity genes with clear mechanisms of synthetic lethality based on multi-omics biomass characterization. We evaluated the performance of the two important prediction submodules in the model through tenfold cross-validation, with the gene expression compensation submodule identifying 18685 predicted pairs with an AUC of 0.937 and the gene co-regulation submodule identifying 194497 predicted pairs with an AUC of 0.879. Ultimately, we combined the intersection of the classification results of the two submodules and identified 7277 potential SL pairs (Supplementary Table S1).

**FIGURE 2:**
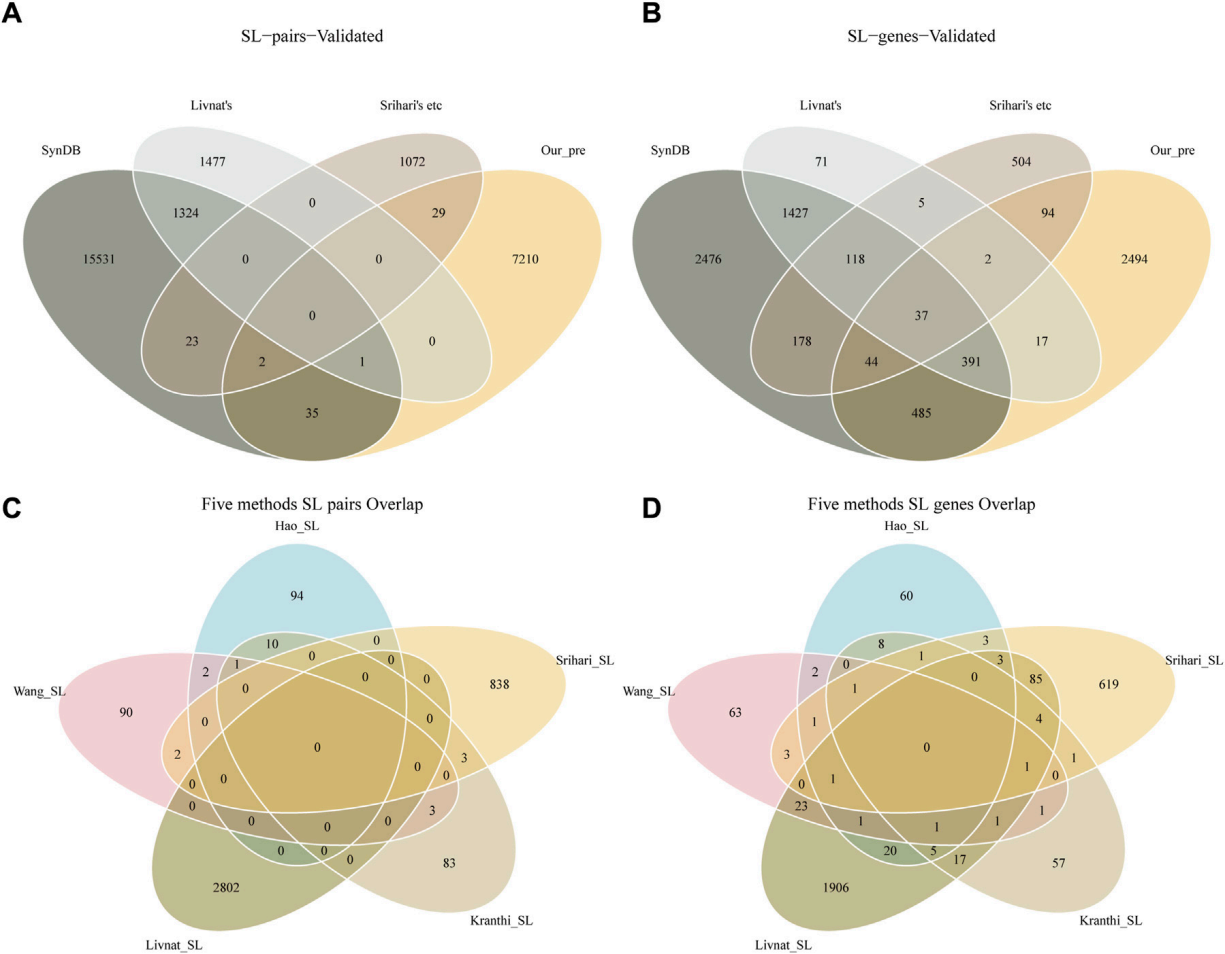
SL genes identified by different methods. **(A)** SL pairs overlap with previous findings. **(B)** Single SL gene overlaps with previous findings. **(C)** The previous five methods of SL pairs overlap with each other. **(D)** The previous five methods of SL genes overlap with each other. SynDB is the SL database SynLethDB records 16916 SL pairs and 5157 genes. Livnat’s is the 2802 SL pairs predicted by Livnat. Srihari’s etc includes the 100 SL pairs predicted by Kranthi, 98 SL pairs predicted by Wang, 843 SL pairs predicted by Srihari and 107 SL pairs predicted by Hao. A total of 3928 unique SL pairs and 3050 genes. Our_pre is the 7277 SL pairs and 3564 genes identified in this study.

Detecting SL pairs in humans is a challenging problem because of the highly evolved, complex, and redundant signaling pathways within human cells. The effects of loss of function caused by gene mutations can often be complemented by parallel pathway signaling. Multiple computational approaches can provide different perspectives on potential SL pairs, such as the correlation of gene expression with mutations, robustness in cancer networks, or co-expression of genes in related biological processes. In our study, we overlapped the 7277 predicted SL pairs with the results from five previous methods (Kranthi (Kranthi et al., 2013), Wang (Wang and Simon, 2013), Srihari (Srihari et al., 2015), Hao (Ye et al., 2016) and Livnat (Jerby-Arnon et al., 2014)) and with the SL pairs recorded in the SynLethDB database (Figures 2A, B). This may suggest that overlapping predictions from different methods may provide more reliable results. Interestingly, we also found no overlap between Livnat’s predictions (Jerby-Arnon et al., 2014) and any of the other four methods. The different characteristics of the input data in these methods may produce bias in SL pairs prediction. In the overlap comparison, our predictions overlap with 38 pairs of SL pairs in SynLethDB, and a total of 32 pairs overlap with the predictions of the previous five-in-one methods. Furthermore, we found little overlap between the results of these five methods (Figures 2C, D). There are some reasons could explain the phenomenon. First, the complexity of the human genome itself is such that many different genes are involved in a pathway that must function to fulfill its biological function. At the same time cancer itself has a complex and highly variable pattern of gene mutations. These factors combine to form complex and variable combinations of synthetic lethal gene pairs, and these large combinatorial spaces adversely affect the replication of research results. Second, the low degree of overlap between different studies may also be due to the differences in their starting points and research methods. Such a view is also supported in the study of Hao et al. (Ye et al., 2016).

### 3.2 Functional analysis of identified SL genes

**FIGURE 3:**
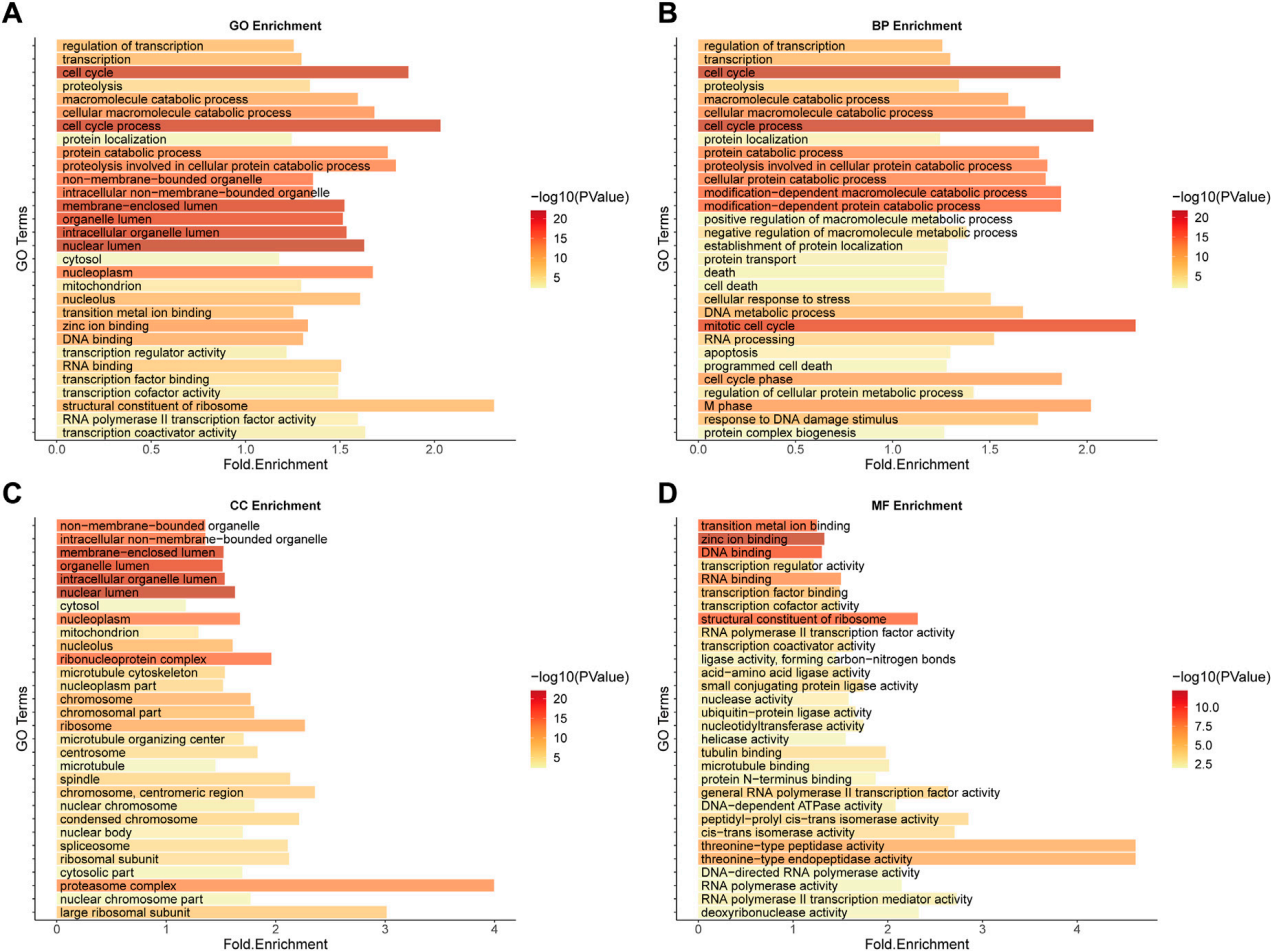
GO Enrichment analysis of potential SL genes in CRC. **(A)** GO enrichment. **(B)** BP enrichment. **(C)** CC enrichment. **(D)** MF enrichment.

The GO functional enrichment analysis of SL genes is closely related to the mechanism and treatment of cancer (Figures 3A–D). For example, as a major disease characterised by malignant abnormal proliferation, the accurate regulation of the cell cycle (GO:0007049-cell cycle, GO:0022402-cell cycle process) is of great importance for the survival and development of organisms, and abnormalities of multiple molecules in the cell cycle could be the cause of cancer (Leake, 1996), not only that, it has been shown that the uptake of cancer nanomedicines changes with the cell cycle stage, illustrating that by developing a combination of cell cycle-specific therapies to achieve a better prognosis for cancer patients should be a focus of cancer drug research (Abouzeid and Torchilin, 2013). In addition, it has been shown that zinc ions, as important cofactors, can efficiently bind to DNA by folding proteins (Cho et al., 1994; Garufi et al., 2015) and that changes in intracellular zinc levels can inactivate p53 function by inducing the protein to adopt a mutant conformation and lose its DNA binding capacity (Meplan et al., 2000; Garufi et al., 2015), which is one of the key oncogenes whose inactivation is important for carcinogenesis. Both zinc-binding (GO:0008270-zinc ion binding) and DNA-binding (GO:0003677-DNA binding) functions are reflected in the functional enrichment results (Supplementary Table S2). The above enrichment analysis results indicate that the function of our predicted SL targets is related to the occurrence and development of CRC and may be potential therapeutic targets.

**FIGURE 4:**
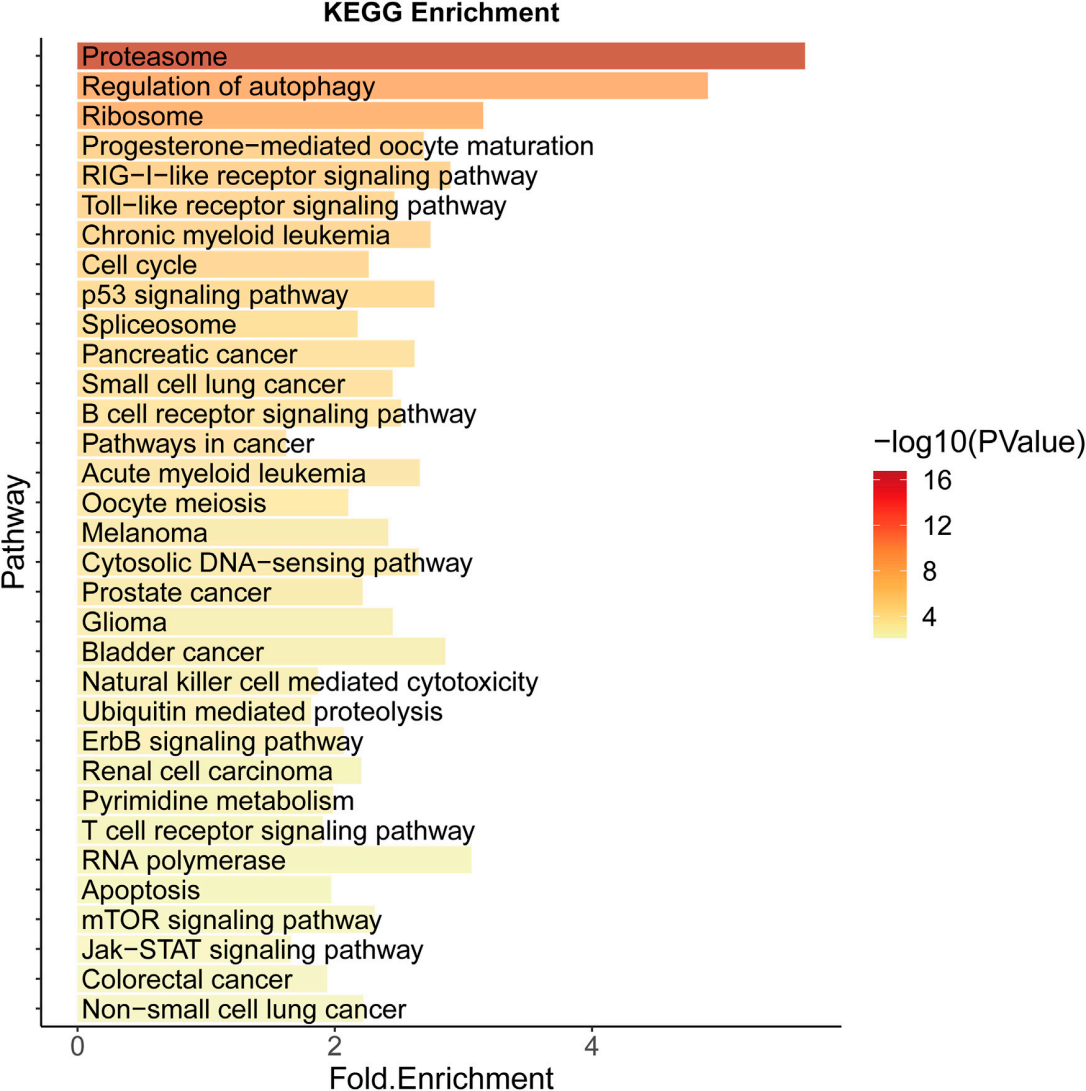
KEGG Enrichment analysis of potential SL genes in CRC.

Significantly enriched pathways in CRC include colorectal cancer (hsa05210), cell cycle (hsa04110), p53 signaling (hsa04115), cancer (hsa05200), and other related pathways, the most important of which is proteasome (hsa03050) (Figure 4). Selective protein degradation plays an important regulatory role in a variety of organismal processes, including the removal of potentially toxic proteins and misfolded proteins to regulate cell cycle progression and gene expression (Glickman and Ciechanover, 2002), and proteasome inhibitors have been shown to have antitumor properties and have been used in clinical settings (Manasanch and Orlowski, 2017), and results have shown that CRC cells can evade proteotoxic stress responses by reducing PSMD5 stimulation of 26S proteasome assembly (Levin et al., 2018).

### 3.3 Identification of potential therapeutic targets for CRC

#### 3.3.1 Identification of CTTs in CRC

**FIGURE 5:**
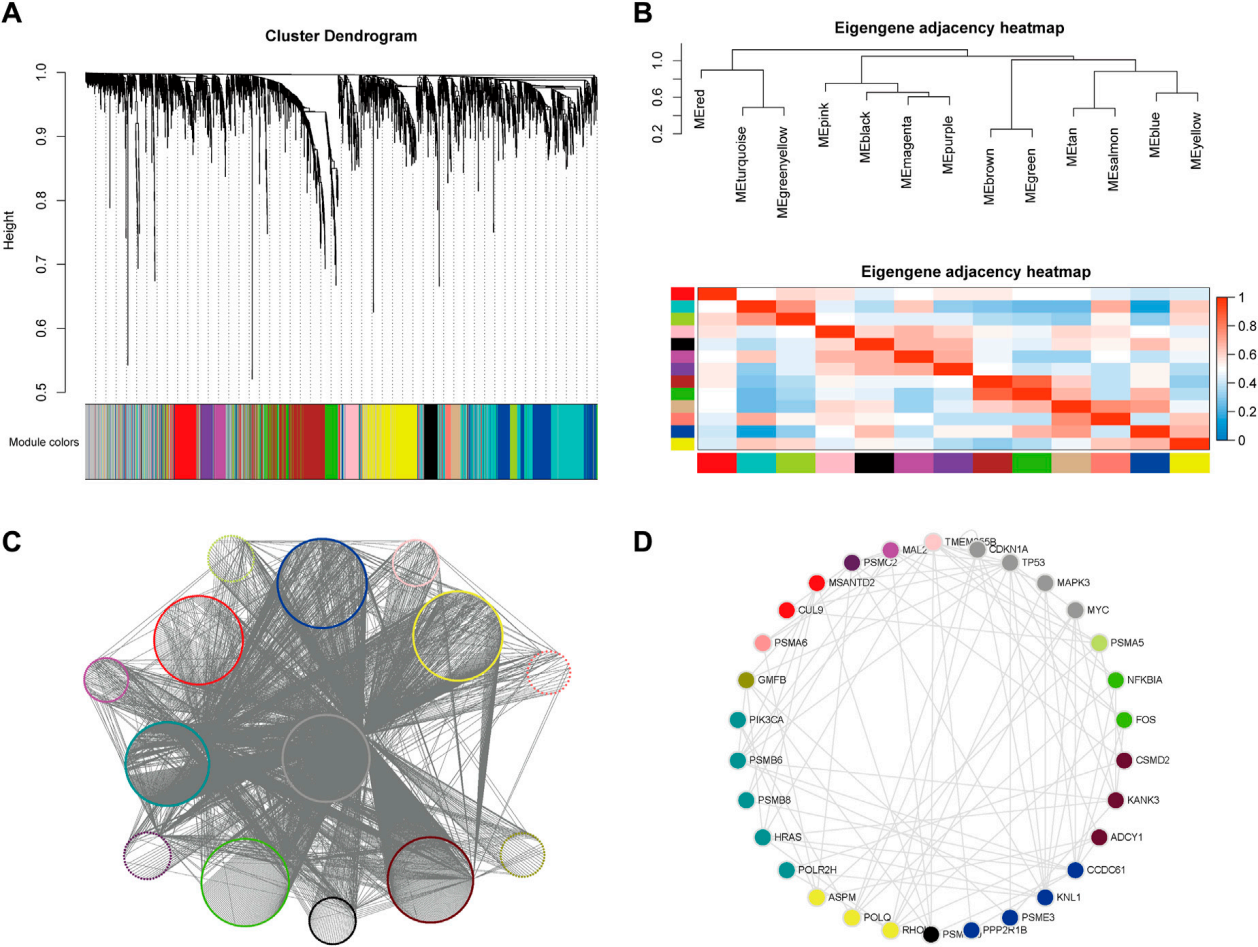
Weighted gene co-expression network analysis (WGCNA) and CTTs modules. **(A)** Hierarchical clustering tree of potential target gene modules for CRC. **(B)** Correlation between gene modules of potential CRC targets. **(C)** The network of WGCNA output modules. **(D)** The top 1% of Hub genes with the highest degree in each module, where the node color represents the module to which the gene is classified.

Alterations in gene function in cancer are often manifested by synergistic and interacting modules between multiple genes, which in themselves can often provide biological mechanistic guidelines for identifying potential therapeutic targets. We therefore performed a further analysis of the previously identified results using WGCNA (Langfelder and Horvath, 2008) (Figure 5). Functional and pathway enrichment analysis was performed for genes in each module (with *p*-value<0.01 and *p*-value<0.05 thresholds, respectively), and several enriched functions and pathways such as RNA binding, cancer-related pathways, and the NF-κB signaling pathway were found to be significantly associated with the cancer-related mechanisms (Supplementary Table S6).

The specific gene regulation and gene expression networks in cancer are the most direct manifestation of its biological state and provide a visual reference for identifying the causative mechanisms and therapeutic approaches to cancer. We therefore analyzed the networks formed by SL pairs in the hope of identifying new centrality genes with numerous SL pairs as promising targets for drug therapy. The classification results were sorted by different module attributes into Cytoscape software, and the top 1% of Hub genes with the highest degree in each module were extracted separately to obtain 30 genes (Supplementary Table S3), namely, PSMD10, PPP2R1B, PSME3, KNL1, CCDC61, ADCY1, CSMD2, KANK3, FOS, NFKBIA, PSMA5, TP53, MYC, CDKN1A, MAPK3, TMEM255B, MAL2, PSMC2, CUL9, MSANTD2, PSMA6, GMFB, PSMB6, PIK3CA, HRAS, POLR2H, PSMB8, ASPM, POLQ and RHOU (Figures 5C, D). Eleven of these genes have been experimentally validated as known CRC-related SL pairs and included in the SynLethDB (Guo et al., 2016) database, namely, PPP2R1B, PSMA5, TP53, MYC, CDKN1A, MAPK3, PSMA6, PSMB6, HRAS, POLR2H, POLQ. In addition, most of the remaining genes were found to be strongly associated with CRC carcinogenesis, metastasis, and prognosis. For example, the trend of significantly high expression of MAL2 in rectal cancer cells was found to be associated with poor patient prognosis (Li et al., 2017), while the expression of the CSMD family was shown to be a predictor of CRC (Zhang and Song, 2014), and KNL1 was associated with reducing apoptosis and promoting proliferation of CRC cells (Bai et al., 2019).

#### 3.3.2 Super-enhancer and ncRNA regulation

**FIGURE 6:**
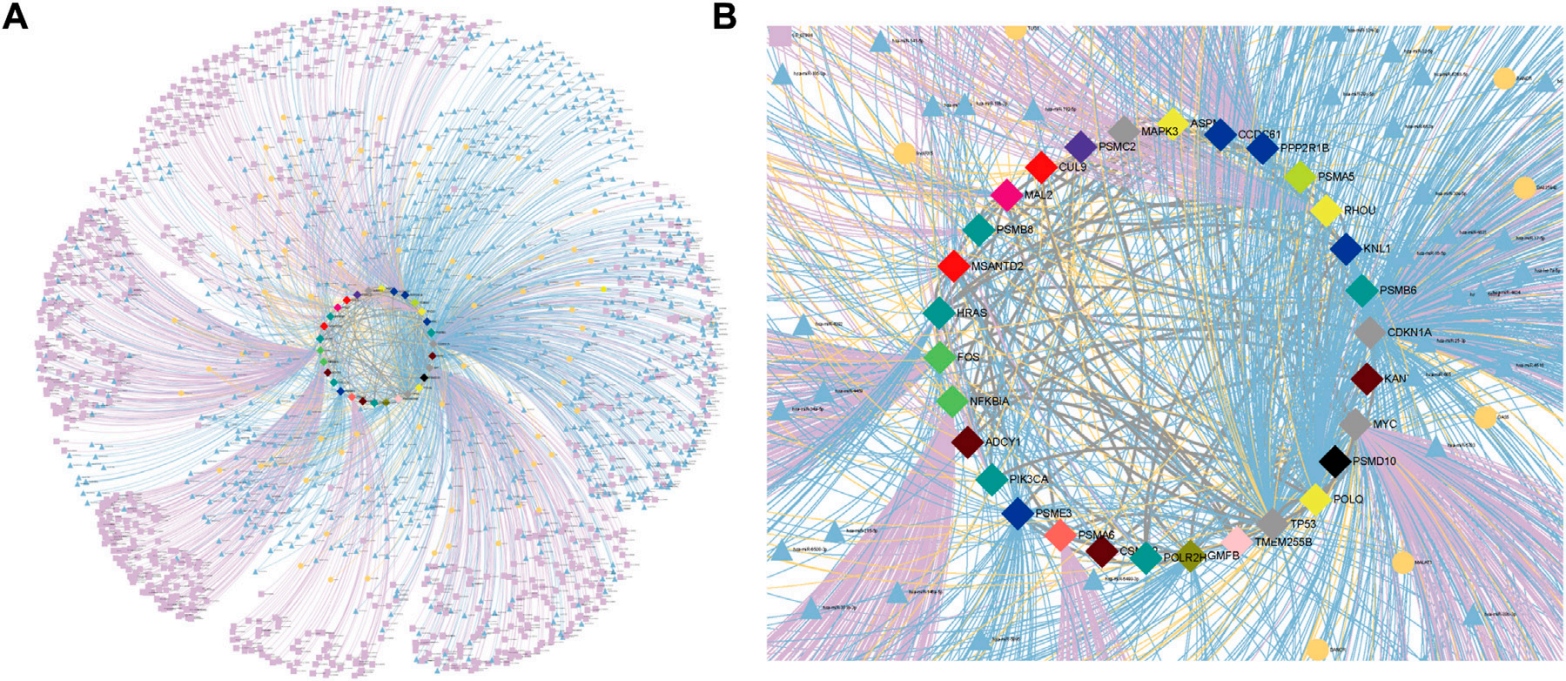
CTTs regulatory relationships for CRC. **(A)** CTTs regulatory network. **(B)** Subgraph of CTTs regulatory network. The regulatory relationships of miRNAs, lncRNAs and super-enhancers for CTTs. The bright blue triangle, bright yellow round and bright purple square nodes and edges represent miRNAs, lncRNAs and super-enhancers targeting important genes and their regulatory relationships on genes, respectively. The central node represents CTTs in CRC, where the node color represents the module to which the gene is classified.

Aberrant regulatory relationships in cancer are often an important factor in its development and progression. Recent studies have shown that super-enhancers (Didych et al., 2015; Thandapani, 2019) and non-coding RNAs (Anastasiadou et al., 2018; Yan and Bu, 2021) often play important roles in cancer-specific regulation. Based on data from dbSUPER (Khan and Zhang, 2016), SEA (Chen et al., 2020), and SEdb (Jiang et al., 2019), their relationship with potential therapeutic targets was specifically analyzed due to their important regulatory roles at the transcriptional and post-transcriptional levels in the SL mechanisms of cancer. The analysis of these hub genes related to miRNAs or lncRNAs regulating CTTs was obtained from miRTarbase (Huang et al., 2020) and LncRNA2Target (Cheng et al., 2019) (Figures 6A, B). These novel regulatory relationships not only provide new insights into the mechanisms of cancer development but also offer new potential avenues for cancer treatment (Supplementary Table S3).

#### 3.3.3 Drug-target interaction network

**FIGURE 7:**
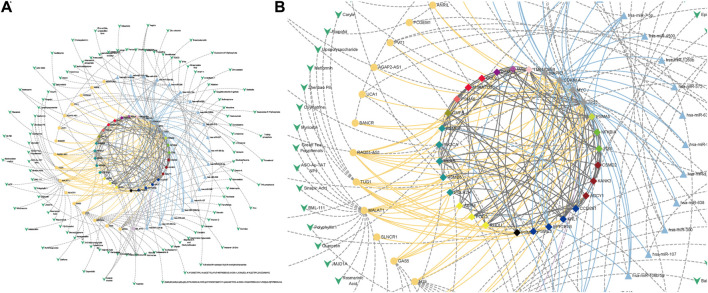
Drug-target interaction in CRC. **(A)** Drug-target interaction network. **(B)** Subgraph of drug-target interaction network. The green V-shaped nodes represent drugs, and the grey dotted edges represent the regulatory effects of drugs. The bright blue, bright yellow and bright purple nodes and edges represent miRNAs, lncRNAs and super-enhancers targeting Hub genes and their regulatory relationships with genes, respectively. The central node represents the node of CTTs in CRC.

Based on our identified CTTs and the regulatory relationships at different regulatory levels, we have screened and identified potential therapeutic drugs for CRC. These identified targets and drugs have the natural advantage of reaching a wider patient population due to their large number of SL partners in a complex SL network. We used the drug target database and literature data to find therapeutic drugs against potential target genes themselves or gene regulatory elements and to generate drug-target interaction maps (Figures 7A, B). For example, Isolinderalactone targets hsa-miR-30c-5p (miRNA) which regulates four CTT genes (TP53, MYC, POLQ and PPP2R1B) and Corylin targets RAD51-AS1 (lncRNA) which regulates eleven CTT genes (PSMD10, PSME3, FOS, NFKBIA, PSMA5, TP53, MYC, PSMC2, CUL9, PSMA6 and PSMB6). Kwak et al. confirmed that Isolinderalactone can induce ROS-mediated apoptosis through the JNK/p38 MAPK signaling pathway, thereby exerting an anticancer effect in CRC Ox-sensitive and OxR cells (Kwak et al., 2022). Yang et al. found that Corylin could significantly reduce the viability of human CRC cells and stimulate apoptosis in a dose-dependent manner (Yang et al., 2021). By targeting these regulatory elements, these CTTs can be regulated. Meantime, for the ncRNA elements, we can only use one drug to target multiple targets enlarging the therapeutic effect. In addition, the modulation of potential target genes by a variety of drugs targeting other cancers or diseases was also identified (Supplementary Table S3 and Supplementary Table S6), which is also important for drug repositioning and guiding clinical drug design for the treatment of CRC.

#### 3.3.4 Mutation of CTTs in CRC

**FIGURE 8:**
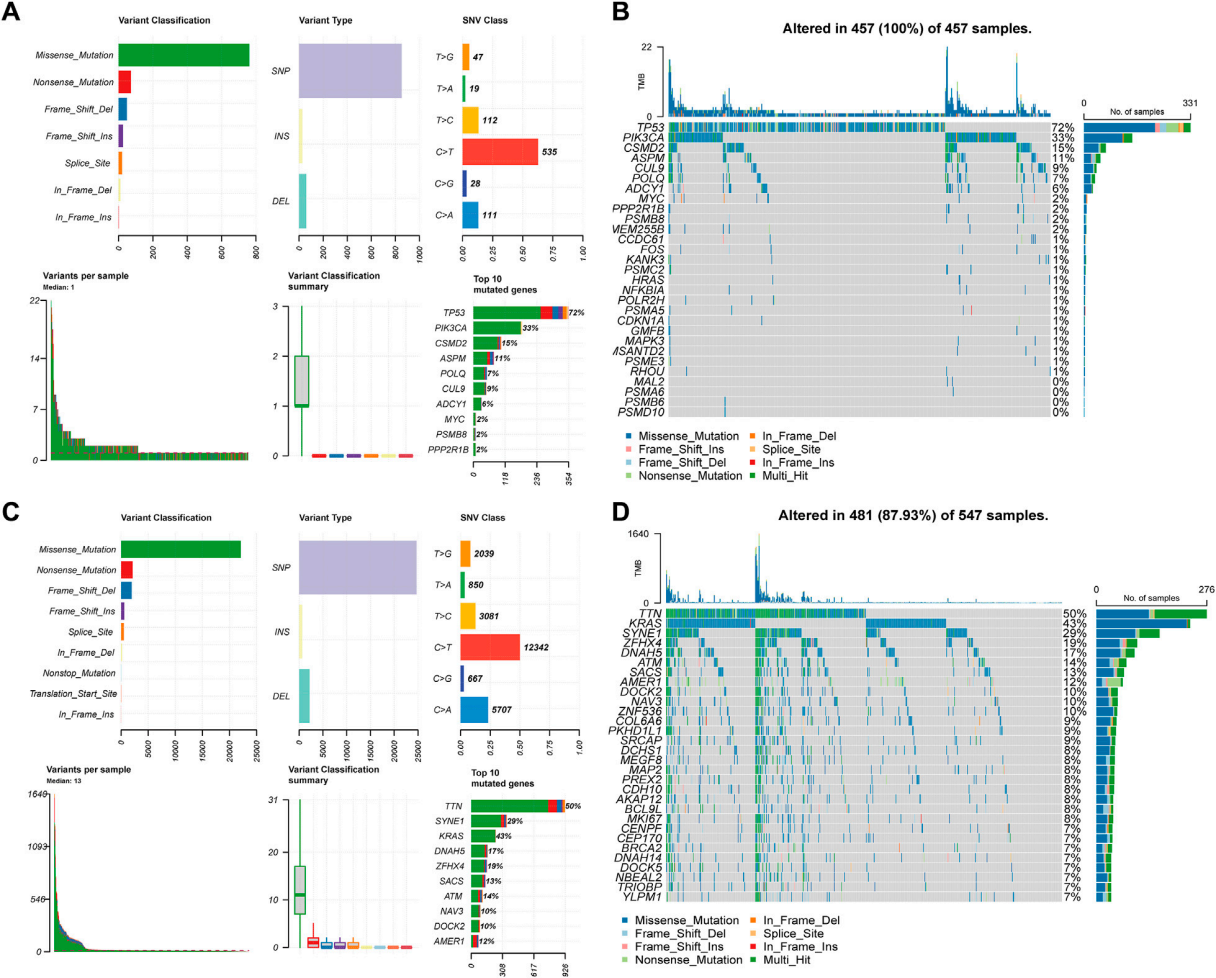
Landscape of somatic mutations of CTTs and non-CTTs SL genes of CRC. **(A) (C)** The distribution of variant classification, variant type, and SNV class present of CTTs and non-CTTs SL genes of CRC. The mutation load of each sample (variant classification type), and the stacked bar graph show the top ten mutated genes. **(B) (D)** Oncoplot and waterfall plot showing the somatic landscape of CTTs and non-CTTs SL genes mutated in CRC.

When a gene is mutated, its SL partner gene loses function through mutation or inhibition, leading to synthetic lethality and cancer cell death. We analyzed CTTs as therapeutic targets and non-CTTs SL genes as somatic mutations in CRC (Figure 8). For 30 CTTs, the variant classification could be divided into seven types, among which missense mutations accounted for the majority. The predominant SNV class was C>T. TP53, PIK3CA, CSMD2, ASPM, POLQ, CUL9, ADCY1, MYC, PSMB8, and PPP2R1B were identified as the most significantly mutated genes (Figure 8A). For non-CTTs SL genes, the variant classification can be divided into nine types, among which missense mutations are also the majority. The predominant SNV class was also C>T. Furthermore, the non-CTTs SL genes in CRC, TTN, SYNE1, KRAS, DNAH5, ZFHX4, SACS, ATM, NAV3, DOCK2 and AMER1 were identified as the most significantly mutated genes (Figure 8C). According to the mutations of 30 CTTs and non-CTTs SL genes in CRC, the TMB of non-CTTs SL genes in some patients with CRC contained a maximum of 1640 mutations, while in patients with CTTs, only 22 mutations were included (Figures 8B, D). Because it is impossible to design drugs one by one for diverse mutation targets, the heterogeneity of cancer poses a huge challenge to targeted therapy for different gene mutations. The method proposed in this study to identify CTTs as therapeutic targets overcomes this shortcoming to some extent. For our predicted result 7277 SL pairs, targeting the 30 CTTs genes could have 4739 interactions result in synthetic lethality and the CTTs have mutation in all CRC patients (Figure 8B). Therefore, although there are different cancer mutations in different patient populations, drugs targeting these 30 CTTs have enormous potential to kill these different mutated cancer cells based on the SL mechanism, thereby achieving the goal of expanding the target treatment of cancer beneficiaries.

In conclusion, CTTs and their partner SL genes cover most of the mutation types in CRC and have a higher level of mutations, which further indicates that the CTTs we found may have a wide range of applicability in CRC.

### 3.4 CTTs and potential drugs identification in other cancers

#### 3.4.1 CTTs and potential drugs for lung cancer

**FIGURE 9:**
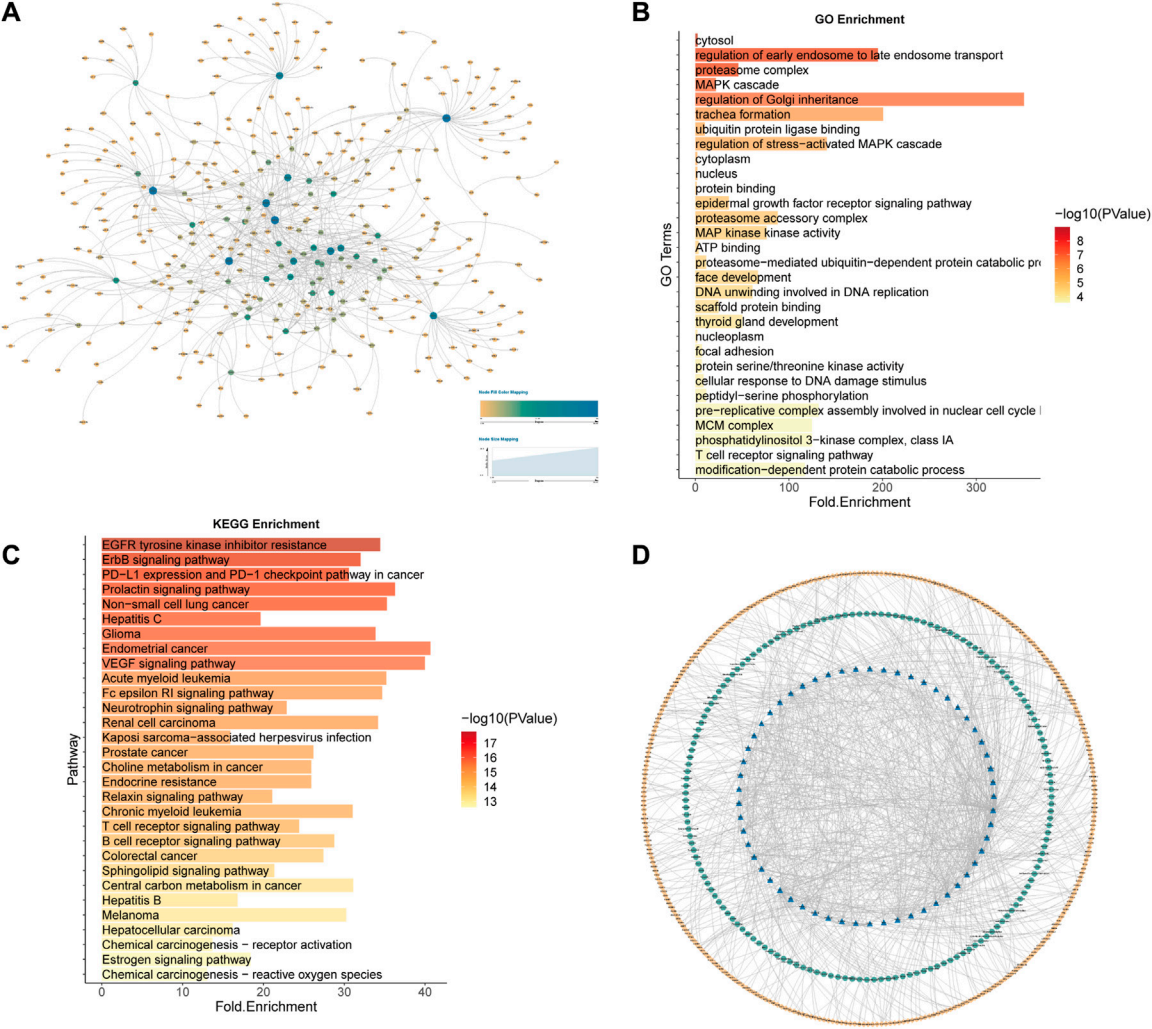
Lung cancer CTTs recognition and corresponding therapeutic drugs screening. **(A)** The network of 824 SL pairs of lung cancer. **(B)** GO enrichment analysis of the 57 CTTs genes. **(C)** KEGG enrichment analysis of the 57 CTTs genes. **(D)**The network of CTTs and targeted drugs. The blue nodes represent CTTs in lung cancer, the green node is targeting CTTs drugs and the yellow node is other SL genes interacting with CTTs.

For the 158 lung cancer samples (LUAD and LUSC) in TCGA, the identification model was constructed using the same data processing method (AUC = 0.96252). The potential 2901 lung cancer SL pairs were predicted, of which 2119 SL pairs came from Wilcoxon rank sum test workflow (*p*-value<0.05), and 1101 SL pairs came from Pearson correlation coefficient test workflow (*p*-value<0.05). The intersection of the two workflows resulted in 824 SL pairs (Figure 9A). Then 57 genes as CTTs were obtained by statistical screening and network analysis (19 of which were verified in SynLethDB) and 178 SL pairs. Functional and pathway enrichment analyses of the 57 CTTs confirmed their closely related enrichment functions to cancer mechanisms and therapeutic approaches (Figures 9B, C).

Combined with the known lung cancer SL genes recorded in SynLethDB, including SL pairs recorded in the literature and SL pairs confirmed by experiments, a total of 19 genes (33.3% of the 57 CTTs genes) were confirmed to be lung cancer-related SL genes, which were DSG2, ADSL, MCM2, MCM4, MCM6, DSP, PARP1, EGFR, KRAS, PIK3CA, MAPK1, UBC, MAPK3, POLR2E, PSMC3, RELA, PSMD6, RBX1, PSMA2.

We also validated them with the listed drugs in Drugbank (Wishart et al., 2018) and TTD (Wang et al., 2020) databases and found that 179 drugs targeting core targets in lung cancer (Figure 9D), were mapped as drug-target interactions network and found that many drugs targeting genes such as EGFR, SRC, PARP1, MAPK1, and FKBP1A, among which EGFR is the epidermal growth factor receptor and high or abnormal expression of EGFR has been confirmed in many solid tumors. Most of the remaining genes are also related to signaling pathways associated with tumor cell proliferation or apoptosis. The data corresponding to cancer changes after cell line administration was obtained from the GDSC (Yang et al., 2013) database and the data of administration belonging to lung cancer cell lines were screened for a total of 181 drugs, of which a total of 13 overlap with our drugs mined through CTTs. Gefitinib, Erlotinib, Lapatinib, Trametinib, Afatinib, and Osimertinib are tyrosine kinase inhibitors, that have been used in the treatment of non-small cell lung cancer and all of which target EGFR (Supplementary Table S4 and Supplementary Table S6).

#### 3.4.2 CTTs and potential drugs for kidney cancer

**FIGURE 10:**
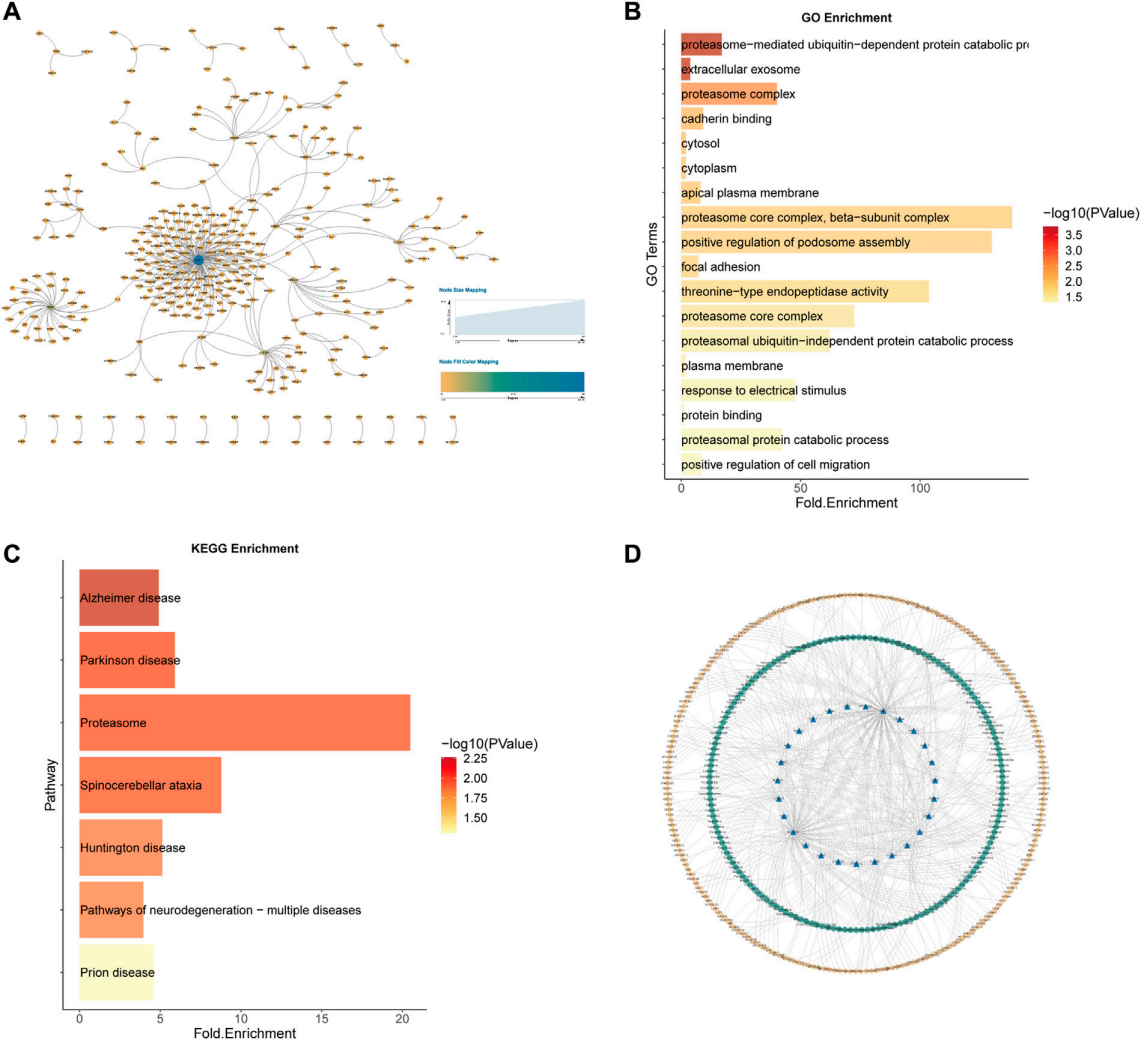
Kidney cancer CTTs recognition and corresponding therapeutic drugs screening. **(A)** The network of 341 SL pairs of kidney cancer. **(B)** GO enrichment analysis of the 27 CTTs genes. **(C)** KEGG enrichment analysis of the 27 CTTs genes. **(D)** The network of CTTs and targeted drugs. The blue nodes represent CTTs in kidney cancer, the green node is targeting CTTs drugs and the yellow node is other SL genes interacting with CTTs.

Similarly, for the 584 kidney cancer samples (KICH, KIRP and KIRC) in TCGA, we predicted the potential 7212 SL pairs for kidney cancer (AUC = 0.8964497), with a total of 1118 SL pairs by Wilcoxon rank sum test (*p*-value<0.05) and 5219 SL pairs by Pearson correlation coefficient test (*p*-value<0.05). The intersection of the two statistical methods yielded 341 SL pairs (Figure 10A). 27 genes as CTTs were obtained by statistical screening and network analysis (eight of them appeared in the previous study results of SL gene pairs of renal cell carcinoma), and functional and pathway enrichment analysis was performed on these 27 CTTs genes (Figures 10B, C).

These 27 CTTs including 8 genes CTNNA1, PSMB6, PSMD12, SESN2, SLC22A2, UBE2J2, and NAE1 appeared in the SL pairs of renal cell carcinoma in previous literature studies. Among the 341 SL pairs, 5 SL pairs appeared in Ku, A. A. et al. (Ku et al., 2020), which were NRAS and APLP2, NRAS and COL6A1, NRAS and MEF2C, NRAS and MSH2, NRAS and NF1.

We took these 27 CTTs as the final research object to mine the drug target database and search for potential drugs for the treatment of renal cell carcinoma. Based on data from the drug target databases Drugbank (Wishart et al., 2018), ChEMBL (Mendez et al., 2019), PubChem (Kim et al., 2021), DGIdb (Cotto et al., 2018), and GDSC (Yang et al., 2013) on cancer changes corresponding to cell line administration, we screened 195 potential drugs for the renal cell cancer (Figure 10D).

A total of 10 drugs have been identified in the literature that overlap with the CTTs targets we mined, i.e., Penandetil, Bortezomib, Cisplatin, Oxaliplatin, Sorafenib, Dasatinib, Tamoxifen, Leflunomide, Paclitaxel and Cyclophosphamide. In addition, we have found that many drugs can target the gene SLC22A2, which is involved in drug transport across the blood-brain barrier and histamine uptake, among other pathways. Most of the drugs that target this gene are used to treat hypertension, which is involved in the pathogenesis of renal cell carcinoma. These results all confirm at different levels the ability of our approach to identify specific CTTs and potential therapeutic drugs in different cancer types (Supplementary Table S5).

## 4 Discussion and conclusion

Existing targeted cancer therapies suffer from the challenges of small populations of therapeutic targets and susceptibility to drug resistance. Using cancers such as CRC as an example, this study creates a general approach to identify potential therapeutic targets and corresponding drugs by constructing a statistical framework with machine learning models for multi-level cancer-specific characterization, expression network analysis and regulatory level analysis prediction. The main advantages of our proposed strategy over previous studies are as follows.

### 4.1 Multidimensional cancer targeting characteristic data description method based on multi-omics data

For the biological characteristics exhibited by cancer development, an integrated and comprehensive characteristic extraction and representation method based on multi-omics biology big data was established at different levels such as gene expression, epigenetic, genomic variation and different perspectives such as expression regulation and post-transcriptional regulation. The essential characteristics of the synthetic lethal mechanism of cancer were further revealed.

### 4.2 Established a model for identifying core therapeutic targets and drugs

In response to the problems that the previously identified cancer therapeutic targets apply to a small population and are prone to drug resistance, this study constructs a statistical and machine learning model based on the synthetic lethal mechanism of cancer cells, which can describe and discover the core therapeutic targets of cancer at multiple levels. The model can identify novel targets that play a central role in multiple cancer-related disease pathways at the level of gene expression, genetic and genomic variation, and screen for potential therapeutic agents in different dimensions such as gene transcriptional regulation and post-transcriptional regulation, thus greatly expanding the existing theoretical and technical approaches for target identification and drug screening in targeted cancer therapy.

### 4.3 Potential application of our method and results to multiple cancer types

This study validated our method for identifying CTTs and discovering corresponding targeted therapeutic agents in colorectal cancer, lung cancer and kidney cancer, respectively, from multiple perspectives, and these results from the literature of other studies, databases and wet experiments suggest that our target and drug identification model is quite generalizable. Nature of cancers identified by CTTs model Biological characteristics are widely present in different types of cancer and have great potential for application and reference value for therapeutic target discovery in pan-cancer types.

Although we have demonstrated the efficacy of the potential therapeutic targets and corresponding drugs obtained in this study in three types of cancer, experimental validation in more types of cancer is still needed. At the same time, the results of this study may offer new hope for cancers that currently have no good treatment options, such as pancreatic cancer, and this is the goal of our further work. In addition, CTTs are promising therapeutic targets because of their ability to kill cancer cells at multiple levels, but they may also cause more complex side effects, which is an issue that needs attention in clinical practice.

## Data Availability

The datasets presented in this study can be found in online repositories. The names of the repository/repositories and accession number(s) can be found in the article/Supplementary Material.
