# Supplementary material for: Multi-level characteristics recognition of cancer core therapeutic targets and drug screening for a broader patient population
Source: Front Pharmacol. 2023 Nov 23;14:1280099. doi: 10.3389/fphar.2023.1280099 (PMC10701285; doi:10.3389/fphar.2023.1280099)
Supplement: Supplementary file 1 [file DataSheet1.zip › Supplementary Files/Supplementary6.docx]

# Summary

Targeted therapies that aim at specific mutations in cancer cells are becoming the first line of clinical treatment for cancer due to their high efficiency and low side effects. However, the problems of single target, limited size of patient population and drug resistance are becoming a challenge in this field. Synthetic lethality is one of the key mechanisms to achieve targeted anticancer therapy. Its main goal is to trigger cancer cell death by inhibiting SL partner of the spontaneously inactivated gene in cancer cells. However, the specificity of different cancer types has led to the fact that there are still very few known SL pairs and their use in clinical medicine is limited, while current experimental methods for identifying SL pairs remain inefficient, costly, and relatively inaccurate. Therefore, this study amis to construct a machine learning model that considers the impact of multifaceted factors on SLs in cancer and to achieve high accuracy in predicting CTTs with broad therapeutic effects in cancer by optimizing the results of gene expression modularization and network analysis to find drugs that act on or have targeted modulation of genes and to address the lack of clinical drugs. This study provides a theoretical and practical reference for the targeted treatment of different types of cancer (including but not limited to colorectal cancer, lung cancer and kidney cancer).

In this study, we integrated CRC-related genomic, epigenomic and proteomic data to construct a data matrix of characteristic sets from different levels and dimensions of cancer molecular signatures. The description of synthetic lethal mechanisms from multidimensional data enables the acquisition and identification of characteristics for targeted cancer therapies from a new and more comprehensive perspective, which provides a data base for the identification of novel potential therapeutic targets and corresponding drugs. On this basis, we developed a statistical framework and machine learning models to describe and identify the relationship between these characteristics and SL targets to identify targets and drugs with a broad role in CRC. This provides new theories and methods for the identification of potential CTTs and drug screening in different types of cancer. Before constructing a positive set based on known SL pairs in CRC, we first performed a step of statistical screening of this gene collection based on the expression pattern of SL pairs. The purpose of these statistical filtering includes: on the one hand because the SL pair is inactivated at the same time, resulting in cell death, when one of the genes is not expressed enough to be inactivated, the compensatory hyperactivity of its SL partner can be analyzed. On the other hand, SL pairs tend to be involved in the same biological process, so to a large extent these SL pairs genes are co-expressed, and this trend has been demonstrated in cancer by experimental assays that do apply to known SL pairs [1]. Based on integrating multi-omics data and considering characteristics provided by different histologies in several aspects, we took the intersection of the two types of experimental results as a positive training sample, and this further filtering refinement helped our CTTs prediction model to have more reliable results. The CTTs proposed in this study as new potential targets bring together synthetic lethal killing properties from different disease and functional pathways, thus providing a stronger lethality of cancer cells than the usual single synthetic lethal relationship. CTTs also offer a wider range of potentially suitable patients and a lower risk of drug resistance due to their role as core nodes with a wide range of mutant synthetic lethal partners. Then, the functional analysis of the prediction results identified various enriched functions such as the proteasome, cell cycle, DNA binding, p53 signaling, zinc ion binding, transcriptional regulation, etc., most of which are importantly related to cancer mechanism and treatment. In addition, this work further classified the genes predicted by the classifier model based on the similarity of expression patterns among genes in a modular way and performed separate functional and pathway enrichment analyses of genes in each module to investigate the inter-module association, which not only identified multiple enriched functions and pathways with a close relationship to the currently known experimentally proven cancer mechanisms but also found that modules with high modular similarity genes may have similar functions and may be involved in gene product cascade reactions along the same pathway. In this study, not only compounds with conventional binding interactions with CTTs were considered in the drug screening phase, but also potential compounds with modulatory effects on CTTs were further considered. This expansion of the screening dimension not only increases the number of potential small molecule therapeutics but also opens up new possibilities for using regulatory macromolecules such as proteins and nucleic acids as potential drugs.

Although we have demonstrated the effectiveness of the potential therapeutic targets and corresponding drugs obtained in this study in three types of cancer, experimental validation is still needed in more types of cancer. At the same time, the results of this study may offer new hope for cancers that currently have no good treatment options, such as pancreatic cancer, and this is the goal of our further work. In addition, CTTs are promising as therapeutic targets because of their ability to kill cancer cells at multiple levels, but they may also bring about more complex side effects, which is an issue that needs attention in clinical practice.

In conclusion, by integrating gene signature data from different histologies associated with cancer and optimizing gene signature data based on expression signatures of SL pairs, this study further improves the reliability of prediction results by constructing a statistical framework with machine learning models to describe and identify these signature relationships, which is an important reference for achieving drug repositioning and rapid development of cancer-targeted drugs.

# Method

## A machine learning-based framework for identifying potential SL pairs

The samples used to model the identification of potential therapeutic targets in this study were mainly SL interactions related to CRC (Colorectal Cancer) from the SynLethDB database. The expression of CRC-associated SL pairs was screened for both the Wilcoxon rank sum test [2, 3] based on gene expression and co-expression[4, 5], respectively, based on the expression characteristics of SL pairs mentioned in the statistical approach of DAISY [6] (Figure 1 B).

According to the SL mechanism, the simultaneous functional inactivation of SL paired genes leads to cancer cell death, while the inactivation of one gene allows the other gene to become essential for cell survival and thus produce overexpression to compensate for the loss of function of the inactivated gene product[2, 3]. Therefore, for each pair of genes AB in our study, the Wilcoxon rank sum test was used to determine whether the expression level of gene B was significantly higher when gene A was under-expressed. We first preprocessed the data to remove samples with abnormal gene expression values and calculated the potential SL of all gene combinations for low activity genes with gene expression levels below 10% of the mean expression level of all samples. The Wilcoxon rank sum test of the R package was used to determine whether there was a significant difference between the expression values of gene A and gene B when gene B was lowly expressed. For such a gene in gene pair(A, B), for example, calculate whether |mRNA(A)-mRNA(B)| is significant at a threshold of P-value<0.01, retaining the gene pairs with significantly different expression levels.

Many studies that have obtained experimental confirmation of SL pairs suggest that SL pairs in cancer cells are more likely to be involved in biological processes that are closely related to each other and therefore both are usually co-expressed[4, 5]. Therefore, we determined whether the expression level of gene pair(A, B) were significantly correlated in the co-expression screen, and calculated the Pearson correlation coefficient R of gene pair(A, B) expression values, using R>0.5 and P-value<0.05 as thresholds to retain the significantly co-expressed pairs.

# Result

## Identification of potential therapeutic targets for CRC

Alterations in gene function in cancer are often manifested through synergistic and interacting modules between multiple genes, which by themselves can often provide biological mechanistic guidelines for identifying potential therapeutic targets. We therefore performed further analysis of the previously identified results using the WGCNA [7]. The functional and pathway enrichment analysis was performed for genes in each module (with P-value<0.01 and P-value<0.05 thresholds, respectively), and several enrichment functions and pathways such as RNA binding, cancer-related pathways, and the NF-κB signaling pathway were found to be significantly related to the cancer-related mechanisms (Figure 5). In contrast, the transcriptional nuclear factor NF-κB signaling pathway is activated in response to growth factors, stress signals, and oncoproteins, etc, to promote gene transcription, with IKKβ binding protein (NIBP) serving to enhance cytokine-induced NF-κB activation by enhancing IKKβ kinase activity [8]. High expression of NIBP has been reported in cancer cell lines and tumor tissues [9]. While the functions of 274 genes in the yellow module are mainly related to the cell cycle. In addition, the main enrichment pathway for 306 genes in the brown module is the mitogen-activated protein kinase (MAPK) signaling pathway (P-value=3.088E-4), and it has been reported that the MAPK signaling pathway is involved in many important regulatory networks that predispose to tumorigenesis, mainly including the regulation of proliferation and apoptosis [10]. Moreover, it has been shown that MAPK may be associated with NF-κB activation and that knockdown of NIBP may reduce metastasis in CRC by downregulating the classical NF-κB signaling pathway and by inhibiting ERK and JNK-mediated MAPK signaling [11]. The high modular similarity between modules green and brown leads to the speculation that the study of intergenic expression propensity has an important reference value for the study of intergenic cascade response.

## Drug-target interaction network

Nadroparin, low molecular weight heparin (LMWH), can stop the coagulation pathway by increasing of rate of complex formation of the physio-logical inhibitors of coagulation, i.e. antithrombin-III and the activated clotting enzymes (mainly thrombin and Xa), and is currently used mainly in the prevention and treatment of thromboembolic diseases, and there are also randomized clinical realizations that confirm that LMWHs may have anti-tumor effects in addition to anticoagulant effects[12]. The antiproliferative effect of heparins and its derivatives on the malignant cell lines is achieved by the inhibition of the protein kinase C-dependent signaling pathway activating transcription factors as cFos and cMyc [13]. Furthermore, it has been claimed that the trend of significantly high MAL2 gene expression in rectal cancer tissues may be associated with poor patient prognosis [14], therefore, it is hypothesized that the inhibition of expression of the model-predicted SL pair outcome FOS-MAL2 in this study could potentially inform the design of therapies targeting rectal cancer. In addition, PPP2R1B, one of the tumor suppressors, is located in a chromosomal segment (11q23) that is frequently deleted by mutations in several cancers, including CRC [15]. 5-Fluorouracil (5-FU) can induce apoptosis in cancer cells by blocking DNA replication and has become one of the most widely used drugs in the cancer treatment including CRC, and one study found that miR-587/ PPP2R1B/pAKT/XIAP signaling axis was found to mediate the response of colon cancer cells to 5-FU therapy, and activation of PPP2R1B expression or reduction of miR-587 expression in colon cancer cells could improve the sensitivity of cells to 5-FU therapy [16]. In contrast, CSMD2, a candidate tumor suppressor gene, was shown to be significantly associated with cell differentiation, lymphovascular infiltration, and tumor size, as well as with poorer patient prognosis, and low expression of CSMD family genes was shown to be a predictor of CRC [17] (Figure 7).

### CTTs and potential drugs for lung cancer

The data corresponding to cancer changes after cell line administration was obtained from the GDSC [18] database, and the data of administration belonging to lung cancer cell lines were screened for a total of 181 drugs, of which a total of 13 overlap with our drugs mined through CTTs, namely Gefitinib, Erlotinib, Lapatinib, Trametinib, Afatinib, Olaparib, Osimertinib, Talazoparib, Niraparib, Ulixertinib, Dasatinib, Sorafenib, Rapamycin, of which Gefitinib, Erlotinib, Lapatinib, Trametinib, Afatinib, Osimertinib, all of which are tyrosine kinase inhibitors, have been used in the treatment of non-small cell lung cancer and all of which target EGFR. Olaparib, Talazoparib and Niraparib, all of which target PARP1, have been used to treat various metastatic breast cancers. Ulixertinib is a novel, reversible, ATP-competitive ERK1/2 inhibitor that is currently in clinical trials for the treatment of various tumors and has the potential to become a novel drug for the treatment of lung cancer. Sorafenib is a kinase inhibitor for the treatment of unresectable liver cancer and advanced kidney cancer that targets the EGFR gene and therefore has the potential to become a treatment for lung cancer. These non-lung cancer therapeutic drugs can be therapeutic candidates for the clinical treatment of lung cancer to some extent, but still, need to be proven in clinical trials. (Figure 9)

## Potentially Consistent Genes in Cancer

Furthermore, we applied the same approach to lung and renal cell carcinomas in the TCGA database, effectively predicting potential CTTs with high confidence, notably nine genes, JUN, NF1, NRAS, PIK3CA, PPP2R1B, PSMB6, PSMC3, PSMD7, and PSMD12 were present in all three cancer types, so we hypothesized that SL pairs centered on these nine genes could be present in all other cancer types.

Yogev et al. [19] suggested that JunB and c-Jun are regulators of autophagy whose expression responds to autophagy-inducing signals. Shaulian et al.[20] believed that Jun could be an oncogene or suppressor in a context-dependent manner.

NF1 mutations are important in the acquisition of drug resistance, to BRAF, EGFR inhibitors, tamoxifen, and retinoic acid in melanoma, lung and breast cancers, and neuroblastoma. A high rate of somatic NF1 mutation in cutaneous melanoma, lung cancer, ovarian carcinoma, and glioblastoma which are not usually associated with neurofibromatosis type 1. Somatic NF1 mutations may be critical drivers in multiple cancers[21].

Roughly a fourth of melanoma patients carry activating NRAS mutations, rendering this malignancy, particularly challenging to treat. Both intrinsic and acquired resistance occur in NRAS-driven melanomas once treated with single or combined targeted therapies involving MAPK and CDK4/6 inhibitors and/or checkpoint inhibiting immunotherapy[22].

Mei ZB et al.[23] findings suggest that PIK3CA mutation has neutral prognostic effects on CRC OS and PFS. Evidence was accumulating for the establishment of CRC survival between PIK3CA mutations and patient-specific clinical or molecular profiles.

Tamaki M et al.[24] suggested that the PPP2R1B gene functions as a tumor suppressor gene and acts as a molecular switch that becomes active in response to specific up-stream signals. Upon activation, the gene alters the activities of specific downstream target proteins for the cell cycle regulations and/or metabolism in some colorectal.

Shi CX et al.[25] show PSMB6 and PSMB7, to be essential for multiple myeloma cell survival, this dependency is structural and the upregulation or activating mutation of PSMB5, 6, and 7 confers proteasome inhibitor resistance, while depletion confers sensitivity. These findings support the modulation of PSMB5, PSMB6, or PSMB7 expression as a new therapeutic strategy.

Tzu-Jen Kao et al.[26] discovered that high levels of PSMC1, PSMC3, PSMC4, PSMC5, and PSMC6 transcripts were positively correlated with poor survival, which likely shows their importance in breast cancer development. Collectively, PSMC family members have the potential to be novel and essential prognostic biomarkers for breast cancer development.

Yuanjie Zhao et al.[27]showed that the PSMD7 level was significantly upregulated in breast cancer tissues. PSMD7 expression was closely associated with tumor subtype, tumor size, lymph node invasion, and TNM stage.

Hypoxia-related genes PSMD12 and PSMB6 were found to be independent prognostic predictive markers for glioma by Feng Gao et al.[28].

In conclusion, our results show that the nine genes in the three cancer types all play a very important role in different cancers. They can be used as tumor suppressor genes or oncogenes, prognostic and therapeutic markers, and so on. Therefore, our findings are expected to become new core therapeutic targets.

# Reference

1. Jerby-Arnon L, Pfetzer N, Waldman YY, McGarry L, James D, Shanks E, Seashore-Ludlow B, Weinstock A, Geiger T, Clemons PA *et al*: **Predicting cancer-specific vulnerability via data-driven detection of synthetic lethality**. *Cell* 2014, **158**(5):1199-1209.

2. Barretina J, Caponigro G, Stransky N, Venkatesan K, Margolin AA, Kim S, Wilson CJ, Lehár J, Kryukov GV, Sonkin D *et al*: **The Cancer Cell Line Encyclopedia enables predictive modelling of anticancer drug sensitivity**. *Nature* 2012, **483**(7391):603-607.

3. Beroukhim R, Mermel CH, Porter D, Wei G, Raychaudhuri S, Donovan J, Barretina J, Boehm JS, Dobson J, Urashima M *et al*: **The landscape of somatic copy-number alteration across human cancers**. *Nature* 2010, **463**(7283):899-905.

4. Costanzo M, Baryshnikova A, Bellay J, Kim Y, Spear ED, Sevier CS, Ding H, Koh JL, Toufighi K, Mostafavi S *et al*: **The genetic landscape of a cell**. *Science (New York, NY)* 2010, **327**(5964):425-431.

5. Kelley R, Ideker T: **Systematic interpretation of genetic interactions using protein networks**. *Nat Biotechnol* 2005, **23**(5):561-566.

6. Ryan CJ, Lord CJ, Ashworth A: **DAISY: picking synthetic lethals from cancer genomes**. *Cancer Cell* 2014, **26**(3):306-308.

7. Langfelder P, Horvath S: **WGCNA: an R package for weighted correlation network analysis**. *BMC Bioinformatics* 2008, **9**:559.

8. Hu WH, Pendergast JS, Mo XM, Brambilla R, Bracchi-Ricard V, Li F, Walters WM, Blits B, He L, Schaal SM *et al*: **NIBP, a novel NIK and IKK(beta)-binding protein that enhances NF-(kappa)B activation**. *The Journal of biological chemistry* 2005, **280**(32):29233-29241.

9. Zhang Y, Liu S, Wang H, Yang W, Li F, Yang F, Yu D, Ramsey FV, Tuszyski GP, Hu W: **Elevated NIBP/TRAPPC9 mediates tumorigenesis of cancer cells through NFκB signaling**. *Oncotarget* 2015, **6**(8):6160-6178.

10. Kim EK, Choi EJ: **Compromised MAPK signaling in human diseases: an update**. *Arch Toxicol* 2015, **89**(6):867-882.

11. Qin M, Zhang J, Xu C, Peng P, Tan L, Liu S, Huang J: **Knockdown of NIK and IKKβ-Binding Protein (NIBP) Reduces Colorectal Cancer Metastasis through Down-Regulation of the Canonical NF-κΒ Signaling Pathway and Suppression of MAPK Signaling Mediated through ERK and JNK**. *PloS one* 2017, **12**(1):e0170595.

12. Nagy Z, Turcsik V, Blasko G: **The effect of LMWH (Nadroparin) on tumor progression**. *Pathol Oncol Res* 2009, **15**(4):689-692.

13. Falanga A, Marchetti M: **Heparin in tumor progression and metastatic dissemination**. *Semin Thromb Hemost* 2007, **33**(7):688-694.

14. Li J, Li Y, Liu H, Liu Y, Cui B: **The four-transmembrane protein MAL2 and tumor protein D52 (TPD52) are highly expressed in colorectal cancer and correlated with poor prognosis**. *PloS one* 2017, **12**(5):e0178515.

15. Baysal BE, Willett-Brozick JE, Taschner PE, Dauwerse JG, Devilee P, Devlin B: **A high-resolution integrated map spanning the SDHD gene at 11q23: a 1.1-Mb BAC contig, a partial transcript map and 15 new repeat polymorphisms in a tumour-suppressor region**. *European journal of human genetics : EJHG* 2001, **9**(2):121-129.

16. Zhang Y, Talmon G, Wang J: **MicroRNA-587 antagonizes 5-FU-induced apoptosis and confers drug resistance by regulating PPP2R1B expression in colorectal cancer**. *Cell Death Dis* 2016, **7**(12):e2525.

17. Zhang R, Song C: **Loss of CSMD1 or 2 may contribute to the poor prognosis of colorectal cancer patients**. *Tumour biology : the journal of the International Society for Oncodevelopmental Biology and Medicine* 2014, **35**(5):4419-4423.

18. Yang W, Soares J, Greninger P, Edelman EJ, Lightfoot H, Forbes S, Bindal N, Beare D, Smith JA, Thompson IR *et al*: **Genomics of Drug Sensitivity in Cancer (GDSC): a resource for therapeutic biomarker discovery in cancer cells**. *Nucleic Acids Res* 2013, **41**(Database issue):D955-961.

19. Yogev O, Goldberg R, Anzi S, Yogev O, Shaulian E: **Jun proteins are starvation-regulated inhibitors of autophagy**. *Cancer Res* 2010, **70**(6):2318-2327.

20. Shaulian E: **AP-1--The Jun proteins: Oncogenes or tumor suppressors in disguise?** *Cell Signal* 2010, **22**(6):894-899.

21. Philpott C, Tovell H, Frayling IM, Cooper DN, Upadhyaya M: **The NF1 somatic mutational landscape in sporadic human cancers**. *Human genomics* 2017, **11**(1):13.

22. Randic T, Kozar I, Margue C, Utikal J, Kreis S: **NRAS mutant melanoma: Towards better therapies**. *Cancer Treat Rev* 2021, **99**:102238.

23. Mei ZB, Duan CY, Li CB, Cui L, Ogino S: **Prognostic role of tumor PIK3CA mutation in colorectal cancer: a systematic review and meta-analysis**. *Annals of oncology : official journal of the European Society for Medical Oncology* 2016, **27**(10):1836-1848.

24. Tamaki M, Goi T, Hirono Y, Katayama K, Yamaguchi A: **PPP2R1B gene alterations inhibit interaction of PP2A-Abeta and PP2A-C proteins in colorectal cancers**. *Oncology reports* 2004, **11**(3):655-659.

25. Shi CX, Zhu YX, Bruins LA, Bonolo de Campos C, Stewart W, Braggio E, Stewart AK: **Proteasome Subunits Differentially Control Myeloma Cell Viability and Proteasome Inhibitor Sensitivity**. *Molecular cancer research : MCR* 2020, **18**(10):1453-1464.

26. Kao TJ, Wu CC, Phan NN, Liu YH, Ta HDK, Anuraga G, Wu YF, Lee KH, Chuang JY, Wang CY: **Prognoses and genomic analyses of proteasome 26S subunit, ATPase (PSMC) family genes in clinical breast cancer**. *Aging* 2021, **13**(14):17970.

27. Zhao Y, Yang X, Xu X, Zhang J, Zhang L, Xu H, Miao Z, Li D, Wang S: **Deubiquitinase PSMD7 regulates cell fate and is associated with disease progression in breast cancer**. *American journal of translational research* 2020, **12**(9):5433-5448.

28. Gao F, Wang Z, Gu J, Zhang X, Wang H: **A Hypoxia-Associated Prognostic Gene Signature Risk Model and Prognosis Predictors in Gliomas**. *Frontiers in oncology* 2021, **11**:726794.
